# HALP score and GNRI: Simple and powerful prognostic markers in diffuse large B-cell lymphoma

**DOI:** 10.1097/MD.0000000000042165

**Published:** 2025-05-30

**Authors:** Tugba Cetintepe, Lutfi Cetintepe, Demet Kiper Unal, Kemal Aygun, Serife Solmaz

**Affiliations:** a Department of Hematology, Izmir Katip Celebi University, Ataturk Training and Research Hospital, Izmir, Turkey; b Department of Nephrology, Izmir Katip Celebi University, Ataturk Training and Research Hospital, Izmir, Turkey.

**Keywords:** albumin, cell lymphoma, diffuse large B, geriatric nutritional risk index (GNRI), hemoglobin, lymphocyte, platelet score (HALP)

## Abstract

Diffuse large B cell lymphoma (DLBCL) is the most common subtype of non-Hodgkin lymphoma. It is a heterogeneous group of diseases that may need very aggressive treatment in some cases. Prognostic evaluation is very important in determining the best treatment. However, in some clinics, access to genetically based and expensive diagnostic methods is limited. In this study, we analyzed the prognostic value of Hemoglobin, Albumin, Lymphocyte and Platelet (HALP) Score and Geriatric Nutritional Risk Index (GNRI) in DLBCL patients. 201 Patients with newly diagnosed DLBLC between 2005 and 2022 were retrospectively evaluated. Optimal cutoff points for the HALP score and the GNRI were calculated with the receiver operating characteristic curve analysis. Kaplan–Meier method was used to compare survival and disease-free survival times between HALP score groups. In the receiver operating characteristic curve analysis, the cutoff points were found 26.17 for HALP, and 99.17 for GNRI. Groups with lower HALP scores and lower GNRIs had significantly shorter overall survival and disease-free survival. Univariate analyses showed that lower HALP score, lower GNRI and higher lactic dehydrogenase, Ann-Arbor stage, International Prognostic Index (IPI), National Comprehensive Cancer Network-IPI and Revised-IPI scores were associated with worse survival rates (*P* < .05). According to the results of the multivariate Cox regression model, patients with HALP score ≤ 26.17 (OR: 2.32; 95%CI: 1.31–4.11 *P* = .004) increased the risk of death (*P* = .001, −2 loglikelihood = 506.15). Similarly, a GNRI score ≤ 99.17 was significantly correlated with worse survival outcomes (*P* < .001). HALP score and GNRI score are reliable, simple and easily accessible indices that can be used to predict the prognosis of DLBLC patients. DLBLC patients with low HALP score and low GNRI level may be associated with short survival and early nutritional support should be considered.

## 1. Introduction

Diffuse large B-cell lymphoma (DLBCL) is the most frequently occurring subtype of non-Hodgkin’s lymphoma, accounting for approximately 25% of cases.^[1]^ Its incidence rises with advancing age. It is a morphologically, genetically and behaviorally heterogeneous disease group with a generally aggressive course.^[2–4]^

The revised Ann Arbor system is used for staging.^[5]^ Prognostic evaluation is primarily based on the International Prognostic Index (IPI), which considers factors such as patient age, lactic dehydrogenase levels, Ann Arbor stage, ECOG performance status, and the presence of extranodal disease.^[6]^ With the advent of rituximab, the Revised IPI (R-IPI) was introduced, and in cases with poorer prognosis, the National Comprehensive Cancer Network (NCCN)-IPI is recommended due to its reported superiority.^[7–9]^

Furthermore, molecular and genetic biomarkers provide important information to the clinician in terms of predicting the course of the disease. In 2016, according to the latest classification of the World Health Organization, it was recommended that DLBCLs should be differentiated immunohistochemically as germinal center B-cell-like and activated B-cell-like and DLBCL patients who need more intensive treatment should be identified by measuring MYC and BCL-2, BCL-6 rearrangements.^[2,10]^ However, unfortunately, it is not possible to study these parameters in all hematology clinics and to access the results of these tests in the pretreatment period, especially in developing countries due to financial costs.

Recently, Hemoglobin, Albumin, Lymphocyte and Platelet (HALP) score, which is calculated by hemoglobin, albumin, lymphocyte and platelet counts, has been defined as a new prognostic marker in solid tumors. However, the effect of this scoring system in hematological cancers is not clear in the literature.

Malnutrition and weight loss may be observed in high-grade lymphomas. In 2005, the Geriatric Nutrition Index (GNRI) was first defined by Bouillanne et al.^[11]^ Serum albumin and the ratio between actual body weight (ABW) and ideal body weight (IBW) are used to calculate GNRI.

The aim of this study was to determine the efficacy of HALP and GNRI scores, which are easy to access, inexpensive and can be calculated with examination results, in predicting prognosis in DLBCL.

## 2. Materials and methods

### 2.1. Inclusion–exclusion criteria

The data of 235 patients diagnosed with DLBCL in the hematology clinic in İzmir between January 2005 and April 2022 were retrospectively evaluated. The data of 34 patients with severe comorbidity, active infection, inadequate organ function and receiving anti-inflammatory therapy were excluded because these conditions may affect the inflammatory response. The study included 201 patients over the age of 18 years. The Lugano revised Ann Arbor response criteria were used for DLBCL response to treatment.^[5]^

### 2.2. Data collection

Detailed information of the patients was obtained from the electronic patient record database of the hospital. Demographic information, complete blood count, biochemical parameters, extranodal involvement, bone marrow biopsy, cytogenetic/FISH evaluation at the time of diagnosis of DLBCL were recorded. Reasons for treatment initiation, first and second and third line treatments in patients who needed treatment, treatment responses, disease-free survival times, survival status, and the effects of treatments on overall survival (OS) were recorded.

### 2.3. Data analysis

Using these data, the HALP score was calculated for each patient with DLBCL at the time of diagnosis. The HALP score was calculated according to the following formula: Hemoglobin (g/L) × albumin (g/L) × lymphocytes (/L)/platelets (/L).^[12]^ IPI, R-IPI, NCCN-IPI at the time of administration were calculated.

The GNRI was calculated as follows: 1.489 × serum albumin level (g/L) + 41.7 × [ABW/IBW (kg)]. Calculated ABW, IBW, and last albumin values of the patients just before the start of R-CHOP treatment were used. If the ABW was higher than the IBW, we have considered the ABW/IBW ratio as 1 in our calculations.

### 2.4. Statistical analysis

Statistical analyses were performed using “IBM SPSS Statistics for Windows. Version 25.0 (Statistical Package for the Social Sciences, IBM Corp., Armonk, NY).” Descriptive statistics were expressed as n and % for categorical variables and Mean ± SD for continuous variables is presented as median (interquartile range).

When the data of the study were analyzed in terms of normality assumptions, Independent *t* test, one of the parametric tests, was applied to determine whether there was a significant difference between the continuous variables with Kolmogorov–Smirnov values *P* > .05 and HALP and GNRI score groups. Chi Square test was used to compare categorical variables. Receiver operating characteristic (ROC) analysis results regarding the prediction of HALP and GNRI scores to be ex are given. Kaplan–Meier method was used to compare survival times and disease-free survival times between HALP score groups. Finally, Univariate and Multivariate Cox Regression results of various clinical factors on the risk of death were given. *P* < .05 was considered statistically significant.

## 3. Results

Among the 201 patients included in the study, 108 were male (53.7%) and 93 were female (46.3%) and the median age was 61 years.

According to Ann Arbor staging at the time of diagnosis, 79 patients (39.3%) had early stage (stage I–II) disease and 122 patients (60.7%) had advanced stage (stage III–IV) disease. 41 patients (20.4%) had splenic involvement. According to the IPI score at the time of diagnosis, the number of low-risk patients was 91 (45.2%), low-intermediate-risk patients was 54 (26.9%), high-intermediate-risk patients was 40 (19.9%) and high-risk patients was 16 (8.0%).

All patients received treatment. Treatment selection was based on the patient’s age, performance status, IPI score and dose-intensive treatment availability. While 70% of the patients received RCHOP-21 chemotherapy, the other patients received R-CHOP-like regimens. Patients with central nervous system DLBCL were treated with more aggressive therapies or radiotherapy.

At the interim evaluation at the 4th month after chemotherapy, complete or partial remission was achieved in 169 patients and 32 patients were unresponsive to first-line treatment. 28 patients had salvage treatment in the second line. 12 patients received autologous stem cell transplantation supported high dose chemotherapy.

The cutoff values for HALP score was not uniform in the previous reports for DLBCL. Hence we performed ROC curve analysis for finding optimal cutoff points for the HALP score. The median HALP score was 26.17. Based on the cutoff points for the HALP score, the patients were categorized into the groups as follows: high HALP score group (>26.17), low HALP group (≤26.17). The estimation of HALP value to discriminate the presence of exitus was statistically significant (*P* = .006). In the ROC analysis, the AUC was 0.622; (95% CI, 0.537–0.707). The number of patients with lower HALP scores was 91, and the number of patients with higher HALP scores was 110.

Among the patients with low HALP scores, 44 (48.4%) were female and 47 (51.6%) were male. Among the patients with high HALP scores, 49 (44.5%) were female and 61 (55.5%) were male. The comparison of prognostic scoring, patient characteristics and treatment results of the patients according to HALP scores are summarized in Table 1.

**Table 1:** Comparison of treatment outcomes and patient characteristics according to HALP scores.

Variable	Overall (n = 201)	HALP		*P*
		>26.17 (n = 110)	≤ 26.17 (n = 91)	
Age (years), mean ± SD	58.88 ± 14.73	58.41 ± 14.46	59.45 ± 15.12	.622*
Gender, n(%)				
Male	108 (53.7)	61 (55.5)	47 (51.6)	.590†
Female	93 (46.3)	49 (44.5)	44 (48.4)	
IPI risk, n(%)				
Low	91 (45.2)	62 (56.4)	29 (31.9)	**.001** †
Low-intermediate	54 (26.9)	29 (26.4)	25 (27.5)	
High-intermediate	40 (19.9)	14 (12.7)	26 (28.5)	
High	16 (8.0)	5 (4.5)	11 (12.1)	
NCCN-IPI risk, n(%)				
Low	36 (17.9)	25 (22.7)	11 (12.0)	**<.001** †
Low-intermediate	83 (41.3)	55 (50.1)	28 (30.8)	
High-intermediate	70 (34.8)	26 (23.6)	44 (48.4)	
High	12 (6.0)	4 (3.6)	8 (8.8)	
R-IPI risk, n(%)				
Low	41 (20.4)	29 (26.4)	12 (13.2)	**.007** †
Low-intermediate	104 (51.7)	59 (53.6)	45 (49.5)	
High	56 (27.9)	22 (20.0)	34 (37.3)	
Ann-Arbor clinical-stage n(%)				
I–II	79 (39.3)	55 (50.0)	24 (26.4)	**.001** †
III–IV	122 (60.7)	55 (50.0)	67 (73.6)	
Infiltration of Bone marrow n(%)				
No	173 (86.1)	94 (85.5)	79 (86.8)	.782†
Yes	28 (13.9)	16 (14.5)	12 (13.2)	
Haemoglobin (mean ± SD.g/L)	123.62 ± 22.06	133.02 ± 19.49	112.25 ± 19.56	**<.001** *
Leukocyte (mean ± SD. 10 9/L	8.28 ± 3.29	8.18 ± 2.90	8.40 ± 3.72	.642*
Lymphocyte(mean ± SD.10 9/L)	1.71 ± 0.76	2.13 ± 0.63	1.21 ± 0.60	**<.001** *
Albumine(mean ± SD. g/L)	39.82 ± 5.55	42.39 ± 4.20	36.72 ± 5.42	**<.001** *
Platelet (mean ± SD.10 9/L)	298.36 ± 120.98	262.37 ± 76.50	341.87 ± 148.12	**<.001** *
Lactate dehydrogenase (IU/L)	275.45 ± 216.85	218.32 ± 97.94	344.51 ± 289.95	**<.001** *
Predicted OS(mean months)	70.95 ± 52.45	82.09 ± 52.88	57.49 ± 48.90	**.001** *
DFS(mean months)	61.92 ± 53.97	72.26 ± 54.42	49.42 ± 50.97	**.003** *
Treatment outcome, n(%)				
Response CR/PR	169 (84.1)	98 (89.1)	71 (78.0)	**.033** †
No response	32 (15.9)	12 (10.9)	20 (22.0)	

Bold values indicate statistically significant differences (*P* < .05).

DFS = disease free survival, HALP = hemoglobin, albumin, lymphocyte and platelet, IPI = International Prognostic Index, NCCN IPI = National Comprehensive Cancer Network International Prognostic Index, OS = overall survival, R-IPI = Revised International Prognostic Index.

* Independent *t* test.

† Chi Square test.

As seen in Table 1, a statistically significant difference was found between the scores of HALP and IPI risk groups (*P* = .001), NCCN-IPI (*P* < .001), R-IPI (*P* = .007), Ann-Arbor clinical-stage groups (*P* = .001), and hemoglobin (*P* < .001), Lymphocyte (*P* < .001), Albumin (*P* < .001), PLT (*P* < .001), lactic dehydrogenase (*P* < .001), OS (months) (*P* = .001), disease free survival (DFS) (months) (*P* = .003), Treatment outcome groups (*P* = .033).

**Figure 1.**
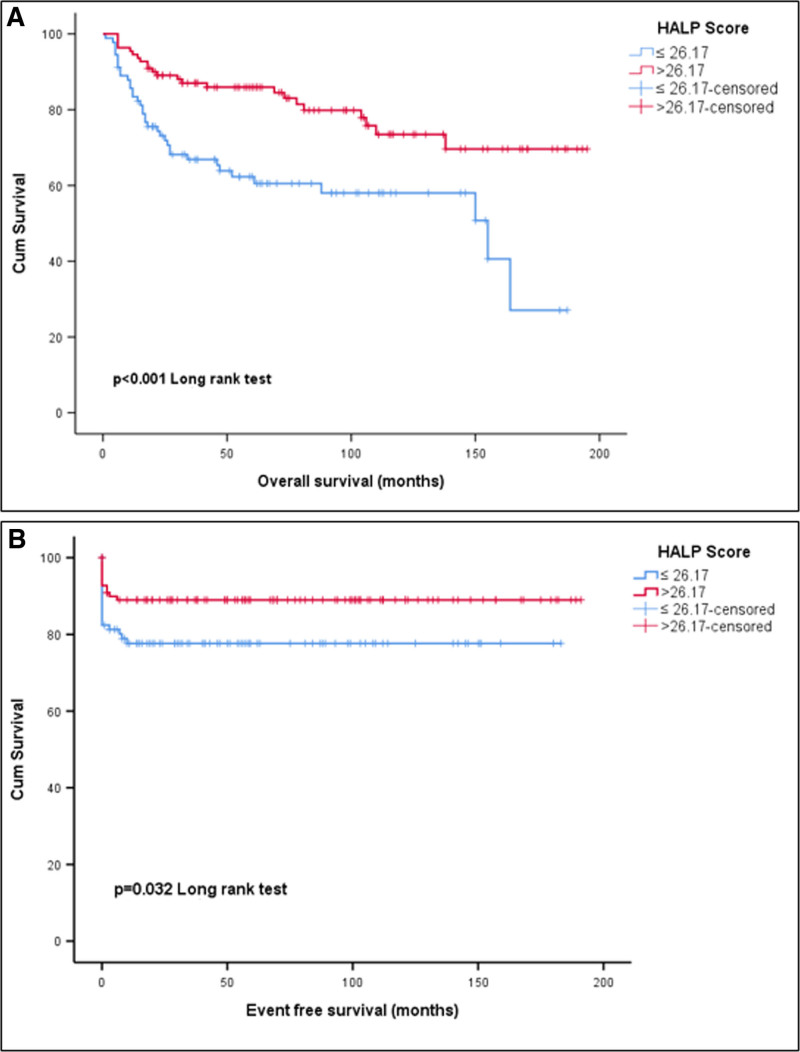
Kaplan–Meier (A) comparison of overall survival times (months) according to HALP scores, (B) comparison of disease-free survival times (months) according to HALP scores. HALP = hemoglobin, albumin, lymphocyte and platelet.

OS (months) and DFS (months) times were shorter in patients with HALP score ≤ 26.17 (Fig. 1). In the posttreatment response evaluation, there was no response in 20 patients with a HALP score ≤ 26.17, while no response was achieved in 12 patients with high HALP scores (*P* = .033).

As seen in Table 2, median OS times (months) according to HALP scores were statistically significant (*P* < .001). Median survival was 155 months (95%CI: 69.47–240.52) in patients with HALP score ≤ 26.17. The 2-year and 5-year survival rates were 73.1% and 62.3%, respectively, in patients with low HALP scores and 89% and 85.9%, respectively, in patients with high HALP scores.

**Table 2:** Overall survival and disease free survival comparisons of patients.

Overall survive (months)	Median (%95 CI)	*P*
HALP		
≤ 26.17	155,00 (69,47–240,52)	<.001
> 26.17	–	
Disease Free Survival (months)	Median (%95 CI)	
HALP	120,00 (73,59–166,40)	
≤ 26.17	–	.032
> 26.17	–	

Kaplan–Meier curve, Long rank test, *P* < .05 is statistically significant.

Median disease-free survival (months) durations according to HALP scores were statistically significant (*P* < .001). Median months could not be reached in patients with HALP score > 26.17 and in patients with HALP score ≤ 26.17. In patients with low HALP score, 5-year disease-free survival was 77.7%, while survival was 89% in patients with high HALP score.

As seen in Table 3, as a result of univariate analyses, low HALP score groups, Ann-Arbor stage, IPI, NCCN-IPI, R-IPI score, and lactic dehydrogenase variables were found statistically significant in terms of mortality risk (*P* < .05). These variables which were found to be significant as a result of univariate analyses were included in the multivariate cox regression model. According to the results of the multivariate Cox regression model, it was determined that HALP score ≤ 26.17 (OR: 2.32; 95%CI: 1.31–4.11 *P* = .004) increased the risk of death (*P* = .001, −2 loglikelihood = 506.15).

**Table 3:** Univariate and multivariate Cox regression results for various clinical variables.

Univariate			Multivariete	*P*
Variables	HR (95%CI)	*P*	HR (95%CI)	
**HALP (Ref:>26.17**)	2.51 (1.49–4.24)	**.001**	2.32 (1.31–4.11)	**.004**
**Ann-Arbor clinical (Ref:I-II**)	2.53 (1.40–4,56)	**.002**	1.44 (0.59–3,47)	.416
**IPI Score (Ref: low**)		**.001**		.402
Low-intermediate	2.14 (1.12–4,09)	**.021**	1.53 (0.65–3,62)	.327
High-intermediate	2.45 (1.21–4,97)	**.012**	1.91 (0.61–5.97)	.266
High	4.78 (2.13–10,72)	**<.001**	3.13 (0.84–11.65)	.084
**NCCN-IPI risk (Ref: low**)		**.002**		.402
Low-intermediate	2.42 (0.92–6,36)	.071	1.73 (0.44–6.78)	.432
High-intermediate	3.21 (1.21–8,51)	**.019**	1.17 (0.24–5,67)	.839
High	7.96 (2.59–24,48)	**<.001**	2.29 (0.39–13,21)	.353
**R-IPI risk (Ref: low**)		**.017**		.924
Low- intermediate	2.12 (0.93–4,84)	**.072**	0.97 (0.29–3,19)	.967
High	3.37 (1.43–7,93)	**.005**	0.83 (0.20–3,41)	.800
**LDH**	1.01 (1.00–1,02)	**.017**	1.00 (0.99–1,01)	.847

*P* = .001, −2 loglikelihood = 554.99. Bold values indicate statistically significant differences (*P* < .05).

HALP = Hemoglobin, Albumin, Lymphocyte and Platelet Score, IPI = International Prognostic Index, LDH = lactic dehydrogenase, NCCN-IPI = National Comprehensive Cancer Network IPI, R-IPI = Revised IPI.

**Figure 2.**
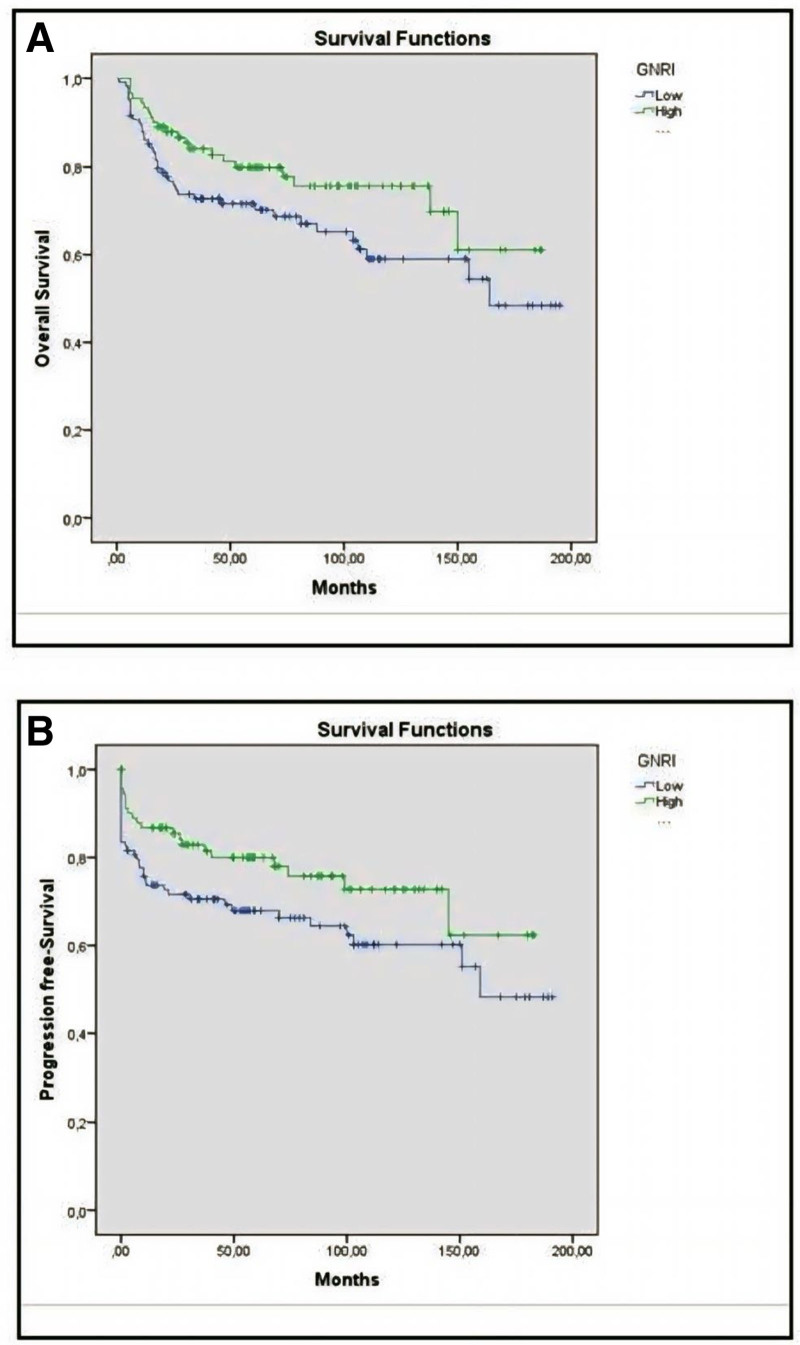
(A) Comparison of overall survival times (months) according to GNRI scores, (B) comparison of progression-free survival times (months) according to GNRI scores. GNRI = Geriatric Nutrition Risk Index.

We performed ROC curve analysis for finding optimal cutoff points for the GNRI score. The median GNRI score was 99.17. Based on the cutoff points for the GNRI score, the patients were categorized into the groups as follows: high GNRI score group (>99.17), low GNRI score group (≤99.16) (with a sensitivity of 63% and specificity of 37%). The number of patients with lower GNRI scores was 109, and the number of patients with higher GNRI scores was 92. The median OS of patients with low GNRI score was 127 ± 8 months (110–143) and median DFS was 120 ± 8.8 (103–137) months, while the median OS of patients with high GNRI score was 143 ± 8 months (127–160) and median DFS was 138 ± 8 (120–155) months (Fig. 2).

## 4. Discussion

The here in presented study aimed to determine the efficacy of HALP score in predicting OS in DLBLC. Multivariate analysis of the study showed that lower HALP score (≤26.17) was a prognostic marker associated with worse survival. Although molecular diagnostic tests provide important information to clinicians in the course of DLBLC, unfortunately, it is not possible to access these parameters in all hematology clinics, especially in developing countries. In this study, a new prognostic marker was calculated for each patient with simple blood values based on basic laboratory measurements as part of the pretreatment evaluation.

Currently, the nutritional status of cancer patients and the role of inflammatory processes in predicting prognosis are being investigated with great interest.^[13–15]^ Serum albumin is a negative acute phase reactant. Nutritional status can be assessed with this molecule synthesized from the liver. Nutritional deficiency, systemic inflammation and increased cytokine release cause a hypercatabolic process and are associated with decreased response against cancer cells. There are many studies in the literature showing that hypoalbumin is associated with negative survival in DLBLC.^[16,17]^

The peripheral blood lymphocyte count is considered an indicator of immunity and lymphocytes play an important role in the defence system against tumors. CD8 + T cells can recognize and eliminate tumor cells through perforin and granzyme B pathways. CD4 + Th cells modulate the tumor microenvironment by secreting cytokines such as IFN-γ, TGF-β, IL-4, IL-5, and IL-6. Low lymphocyte count in newly diagnosed DLBLC patients negatively affects survival and treatment responses.^[18,19]^ Anemia may be present in lymphoproliferative disorders for many reasons including nutritional reasons, hypercatabolic state, bone marrow infiltration. Low hemoglobin is an independent risk factor associated with poor survival in lymphoma patients.^[20,21]^

In consideration of the above data, the HALP score, which is calculated using hemoglobin, lymphocytes, albumin and platelets, is a scoring system that can evaluate both the patient’s immune system and nutritional status in detail. Recently, the correlation between HALP score and prognosis has been evaluated in many solid tumors. Feng et al determined the postoperative HALP score as a potential independent prognostic factor for survival in 355 resectable esophageal squamous cell carcinomas.^[22]^ HALP score was also found to be an independent risk factor in metastatic renal cell carcinoma, metastatic prostate carcinoma and bladder cancer after radical cystectomy.^[23–25]^ In patients over 65 years of age with small cell lung cancer, HALP score before first-line etoposide treatment was significant, whereas higher HALP score after radical surgery in non-small cell lung cancer was associated with longer survival.^[26–28]^

This study showed that survival was shortened in DLBLC with HALP score ≤ 26.17 and this value was an independent risk factor.

The GNRI reflects the nutritional status of the patient as it is a measurement based on serum albumin concentration and the patient’s weight. A low GNRI score is highly associated with malnutrition. GNRI is a simple method that has been established as a strong prognostic factor in some tumors.^[28–32]^

Serum albumin is a simple and valuable marker that can reflect malnutrition and cachexia in cancer patients. Some studies have also shown that weight loss is a poor prognostic marker in DLBCL.

The results of our study showed that DLBCL cases with GNRI score >99.17 were associated with longer survival. In our patients, GNRI score is an independent predictor of survival in accordance with the literature.

This is a study to determine the effect of HALP score and GNRI score on OS in DLBCL. However, the fact that the study was a retrospective and single-center study was a limiting factor.

## 5. Conclusions

HALP score and GNRI score are reliable, simple, easily accessible indices that can be used to predict the prognosis of DLBCL patients. DLBCL patients with low HALP score and low GNRI level may be associated with short survival and early nutritional support should be considered.

## Author contributions

**Conceptualization:** Tugba Cetintepe, Serife Solmaz.

**Data curation:** Tugba Cetintepe, Lutfi Cetintepe.

**Formal analysis:** Tugba Cetintepe, Demet Kiper Unal, Kemal Aygun, Serife Solmaz.

**Funding acquisition:** Tugba Cetintepe, Lutfi Cetintepe.

**Investigation:** Tugba Cetintepe, Serife Solmaz.

**Methodology:** Tugba Cetintepe.

**Project administration:** Tugba Cetintepe, Serife Solmaz.

**Resources:** Tugba Cetintepe.

**Software:** Tugba Cetintepe, Serife Solmaz.

**Supervision:** Tugba Cetintepe, Serife Solmaz.

**Visualization:** Tugba Cetintepe.

**Writing – original draft:** Tugba Cetintepe.

**Writing – review & editing:** Tugba Cetintepe, Lutfi Cetintepe, Demet Kiper Unal, Kemal Aygun, Serife Solmaz.
